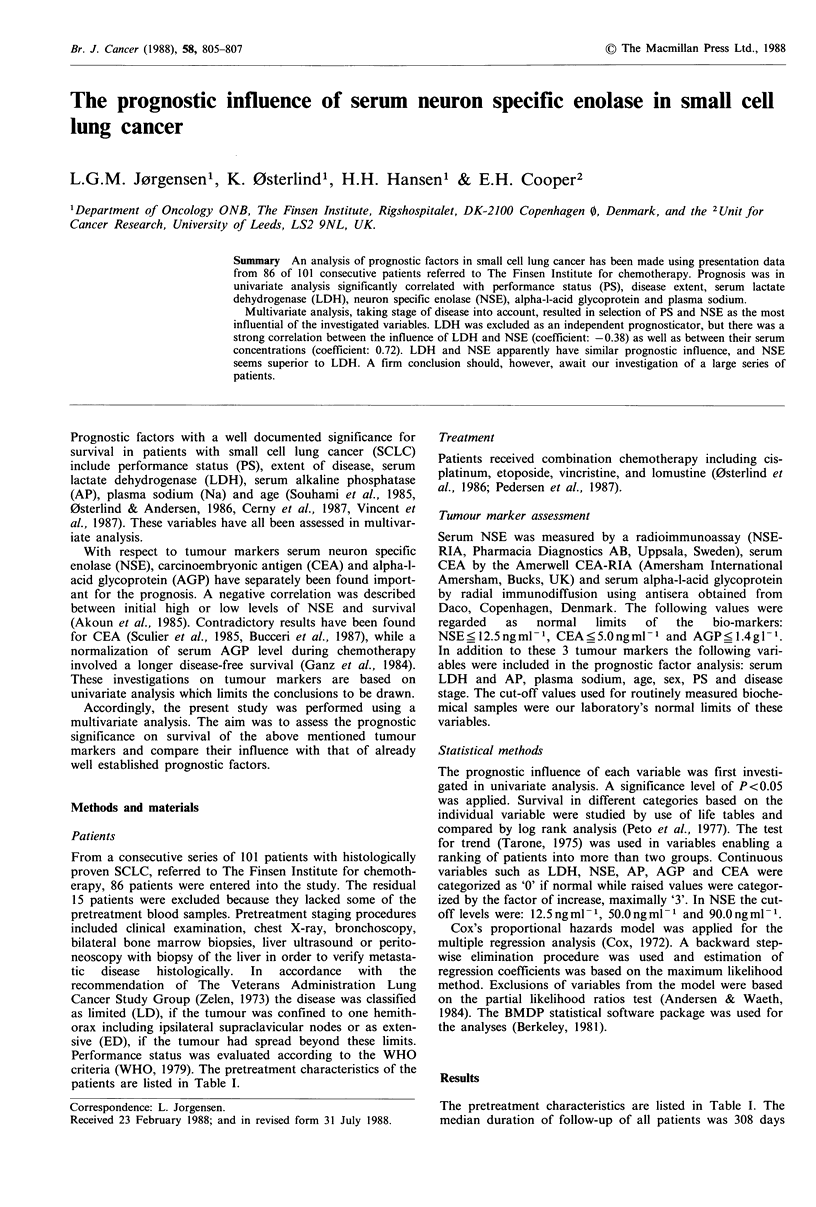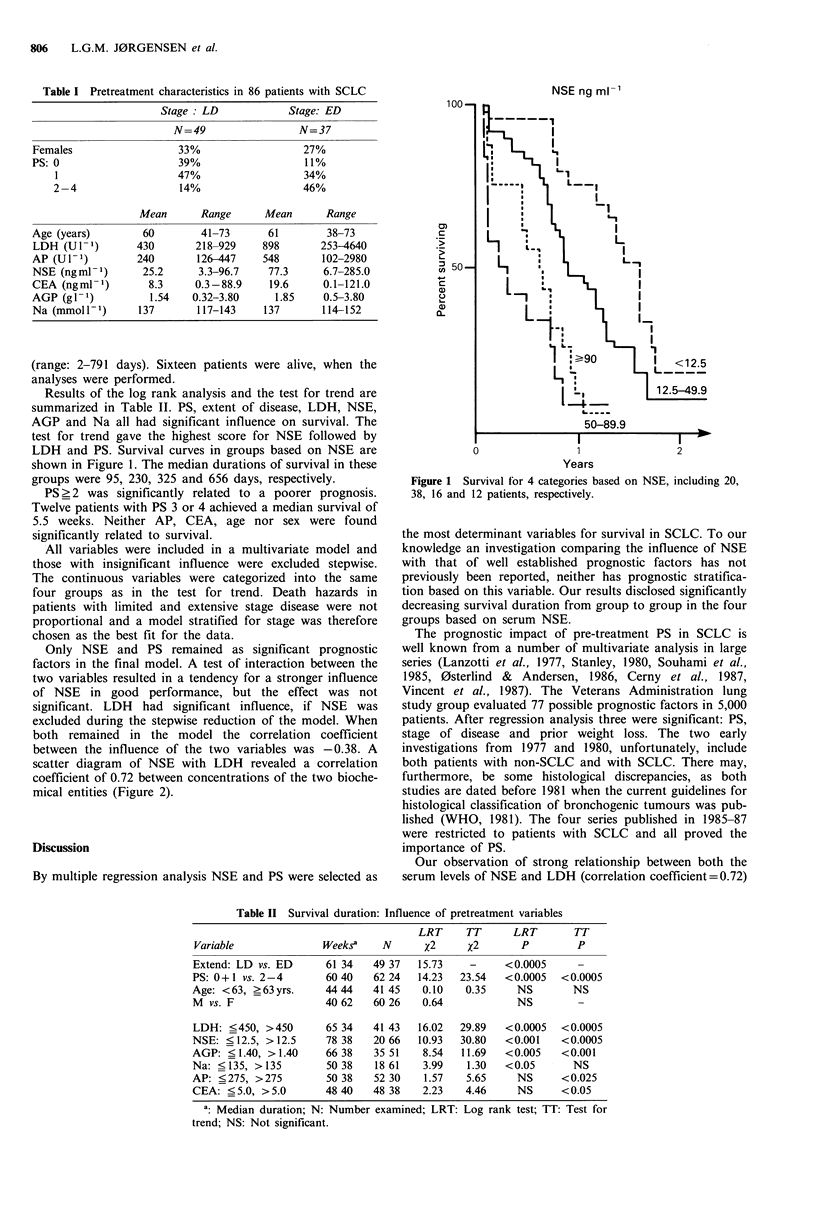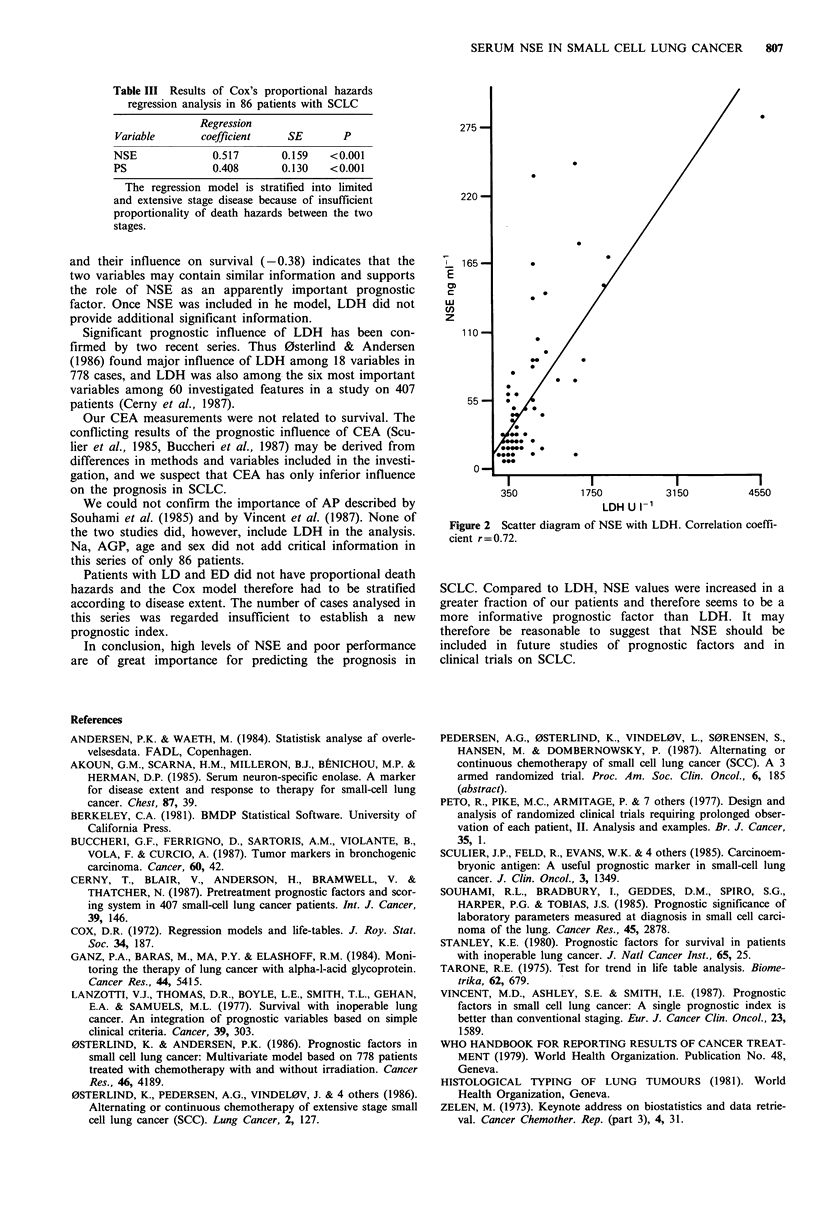# The prognostic influence of serum neuron specific enolase in small cell lung cancer.

**DOI:** 10.1038/bjc.1988.313

**Published:** 1988-12

**Authors:** L. G. Jørgensen, K. Osterlind, H. H. Hansen, E. H. Cooper

**Affiliations:** Department of Oncology ONB, Finsen Institute, Rigshospitalet, Copenhagen, Denmark.

## Abstract

An analysis of prognostic factors in small cell lung cancer has been made using presentation data from 86 of 101 consecutive patients referred to The Finsen Institute for chemotherapy. Prognosis was in univariate analysis significantly correlated with performance status (PS), disease extent, serum lactate dehydrogenase (LDH), neuron specific enolase (NSE), alpha-1-acid glycoprotein and plasma sodium. Multivariate analysis, taking stage of disease into account, resulted in selection of PS and NSE as the most influential of the investigated variables. LDH was excluded as an independent prognosticator, but there was a strong correlation between the influence of LDH and NSE (coefficient: -0.38) as well as between their serum concentrations (coefficient: 0.72). LDH and NSE apparently have similar prognostic influence, and NSE seems superior to LDH. A firm conclusion should, however, await our investigation of a large series of patients.


*Br. J. Cancer* (1988), **58**, 805–807
© The Macmillan Press Ltd., 1988

### The prognostic influence of serum neuron specific enolase in small cell lung cancer

L.G.M. Jørgensen[1], K. Østerlind[1], H.H. Hansen[1] & E.H. Cooper[2]

[1]*Department of Oncology ONB, The Finsen Institute, Rigshospitalet, DK-2100 Copenhagen Ø, Denmark, and the* [2]*Unit for Cancer Research, University of Leeds, LS2 9NL, UK.*

**Summary** An analysis of prognostic factors in small cell lung cancer has been made using presentation data from 86 of 101 consecutive patients referred to The Finsen Institute for chemotherapy. Prognosis was in univariate analysis significantly correlated with performance status (PS), disease extent, serum lactate dehydrogenase (LDH), neuron specific enolase (NSE), alpha-l-acid glycoprotein and plasma sodium.

Multivariate analysis, taking stage of disease into account, resulted in selection of PS and NSE as the most influential of the investigated variables. LDH was excluded as an independent prognosticator, but there was a strong correlation between the influence of LDH and NSE (coefficient: −0.38) as well as between their serum concentrations (coefficient: 0.72). LDH and NSE apparently have similar prognostic influence, and NSE seems superior to LDH. A firm conclusion should, however, await our investigation of a large series of patients.

Prognostic factors with a well documented significance for survival in patients with small cell lung cancer (SCLC) include performance status (PS), extent of disease, serum lactate dehydrogenase (LDH), serum alkaline phosphatase (AP), plasma sodium (Na) and age (Souhami *et al.*, 1985, Østerlind & Andersen, 1986, Cerny *et al.*, 1987, Vincent *et al.*, 1987). These variables have all been assessed in multivariate analysis.

With respect to tumour markers serum neuron specific enolase (NSE), carcinoembryonic antigen (CEA) and alpha-l-acid glycoprotein (AGP) have separately been found important for the prognosis. A negative correlation was described between initial high or low levels of NSE and survival (Akoun *et al.*, 1985). Contradictory results have been found for CEA (Sculier *et al.*, 1985, Bucceri *et al.*, 1987), while a normalization of serum AGP level during chemotherapy involved a longer disease-free survival (Ganz *et al.*, 1984). These investigations on tumour markers are based on univariate analysis which limits the conclusions to be drawn.

Accordingly, the present study was performed using a multivariate analysis. The aim was to assess the prognostic significance on survival of the above mentioned tumour markers and compare their influence with that of already well established prognostic factors.

### Methods and materials

### *Patients*

From a consecutive series of 101 patients with histologically proven SCLC, referred to The Finsen Institute for chemotherapy, 86 patients were entered into the study. The residual 15 patients were excluded because they lacked some of the pretreatment blood samples. Pretreatment staging procedures included clinical examination, chest X-ray, bronchoscopy, bilateral bone marrow biopsies, liver ultrasound or peritoneoscopy with biopsy of the liver in order to verify metastatic disease histologically. In accordance with the recommendation of The Veterans Administration Lung Cancer Study Group (Zelen, 1973) the disease was classified as limited (LD), if the tumour was confined to one hemithorax including ipsilateral supraclavicular nodes or as extensive (ED), if the tumour had spread beyond these limits. Performance status was evaluated according to the WHO criteria (WHO, 1979). The pretreatment characteristics of the patients are listed in Table I.

Correspondence: L. Jorgensen.
Received 23 February 1988; and in revised form 31 July 1988.

### *Treatment*

Patients received combination chemotherapy including cisplatinum, etoposide, vincristine, and lomustine (Østerlind *et al.*, 1986; Pedersen *et al.*, 1987).

### *Tumour marker assessment*

Serum NSE was measured by a radioimmunoassay (NSE-RIA, Pharmacia Diagnostics AB, Uppsala, Sweden), serum CEA by the Amerwell CEA-RIA (Amersham International Amersham, Bucks, UK) and serum alpha-l-acid glycoprotein by radial immunodiffusion using antisera obtained from Daco, Copenhagen, Denmark. The following values were regarded as normal limits of the bio-markers: NSE $\leq 12.5\,ng\,ml^{-1}$, CEA $\leq 5.0\,ng\,ml^{-1}$ and AGP $\leq 1.4\,g\,l^{-1}$. In addition to these 3 tumour markers the following variables were included in the prognostic factor analysis: serum LDH and AP, plasma sodium, age, sex, PS and disease stage. The cut-off values used for routinely measured biochemical samples were our laboratory's normal limits of these variables.

### *Statistical methods*

The prognostic influence of each variable was first investigated in univariate analysis. A significance level of $P<0.05$ was applied. Survival in different categories based on the individual variable were studied by use of life tables and compared by log rank analysis (Peto *et al.*, 1977). The test for trend (Tarone, 1975) was used in variables enabling a ranking of patients into more than two groups. Continuous variables such as LDH, NSE, AP, AGP and CEA were categorized as '0' if normal while raised values were categorized by the factor of increase, maximally '3'. In NSE the cut-off levels were: $12.5\,ng\,ml^{-1}$, $50.0\,ng\,ml^{-1}$ and $90.0\,ng\,ml^{-1}$.

Cox's proportional hazards model was applied for the multiple regression analysis (Cox, 1972). A backward stepwise elimination procedure was used and estimation of regression coefficients was based on the maximum likelihood method. Exclusions of variables from the model were based on the partial likelihood ratios test (Andersen & Waeth, 1984). The BMDP statistical software package was used for the analyses (Berkeley, 1981).

### Results

The pretreatment characteristics are listed in Table I. The median duration of follow-up of all patients was 308 days

**Table I** Pretreatment characteristics in 86 patients with SCLC

| | *Stage : LD* | | *Stage: ED* | |
|---|---|---|---|---|
| | *N=49* | | *N=37* | |
| Females | 33% | | 27% | |
| PS: 0 | 39% | | 11% | |
| 1 | 47% | | 34% | |
| 2–4 | 14% | | 46% | |
| | *Mean* | *Range* | *Mean* | *Range* |
| Age (years) | 60 | 41–73 | 61 | 38–73 |
| LDH ($U l^{-1}$) | 430 | 218–929 | 898 | 253–4640 |
| AP ($U l^{-1}$) | 240 | 126–447 | 548 | 102–2980 |
| NSE ($ng ml^{-1}$) | 25.2 | 3.3–96.7 | 77.3 | 6.7–285.0 |
| CEA ($ng ml^{-1}$) | 8.3 | 0.3–88.9 | 19.6 | 0.1–121.0 |
| AGP ($g l^{-1}$) | 1.54 | 0.32–3.80 | 1.85 | 0.5–3.80 |
| Na ($mmol l^{-1}$) | 137 | 117–143 | 137 | 114–152 |

(range: 2–791 days). Sixteen patients were alive, when the analyses were performed.

Results of the log rank analysis and the test for trend are summarized in Table II. PS, extent of disease, LDH, NSE, AGP and Na all had significant influence on survival. The test for trend gave the highest score for NSE followed by LDH and PS. Survival curves in groups based on NSE are shown in Figure 1. The median durations of survival in these groups were 95, 230, 325 and 656 days, respectively.

PS$\geq$2 was significantly related to a poorer prognosis. Twelve patients with PS 3 or 4 achieved a median survival of 5.5 weeks. Neither AP, CEA, age nor sex were found significantly related to survival.

All variables were included in a multivariate model and those with insignificant influence were excluded stepwise. The continuous variables were categorized into the same four groups as in the test for trend. Death hazards in patients with limited and extensive stage disease were not proportional and a model stratified for stage was therefore chosen as the best fit for the data.

Only NSE and PS remained as significant prognostic factors in the final model. A test of interaction between the two variables resulted in a tendency for a stronger influence of NSE in good performance, but the effect was not significant. LDH had significant influence, if NSE was excluded during the stepwise reduction of the model. When both remained in the model the correlation coefficient between the influence of the two variables was −0.38. A scatter diagram of NSE with LDH revealed a correlation coefficient of 0.72 between concentrations of the two biochemical entities (Figure 2).

### Discussion

By multiple regression analysis NSE and PS were selected as the most determinant variables for survival in SCLC. To our knowledge an investigation comparing the influence of NSE with that of well established prognostic factors has not previously been reported, neither has prognostic stratification based on this variable. Our results disclosed significantly decreasing survival duration from group to group in the four groups based on serum NSE.

**Figure 1** Survival for 4 categories based on NSE, including 20, 38, 16 and 12 patients, respectively.

The prognostic impact of pre-treatment PS in SCLC is well known from a number of multivariate analysis in large series (Lanzotti *et al.*, 1977, Stanley, 1980, Souhami *et al.*, 1985, Østerlind & Andersen, 1986, Cerny *et al.*, 1987, Vincent *et al.*, 1987). The Veterans Administration lung study group evaluated 77 possible prognostic factors in 5,000 patients. After regression analysis three were significant: PS, stage of disease and prior weight loss. The two early investigations from 1977 and 1980, unfortunately, include both patients with non-SCLC and with SCLC. There may, furthermore, be some histological discrepancies, as both studies are dated before 1981 when the current guidelines for histological classification of bronchogenic tumours was published (WHO, 1981). The four series published in 1985–87 were restricted to patients with SCLC and all proved the importance of PS.

Our observation of strong relationship between both the serum levels of NSE and LDH (correlation coefficient = 0.72)

**Table II** Survival duration: Influence of pretreatment variables

| *Variable* | *Weeks*[a] | *N* | *LRT* $\chi 2$ | *TT* $\chi 2$ | *LRT* *P* | *TT* *P* |
|---|---|---|---|---|---|---|
| Extend: LD *vs.* ED | 61 34 | 49 37 | 15.73 | – | <0.0005 | – |
| PS: 0+1 *vs.* 2–4 | 60 40 | 62 24 | 14.23 | 23.54 | <0.0005 | <0.0005 |
| Age: <63, ≥63 yrs. | 44 44 | 41 45 | 0.10 | 0.35 | NS | NS |
| M *vs.* F | 40 62 | 60 26 | 0.64 | | NS | – |
| LDH: ≤450, >450 | 65 34 | 41 43 | 16.02 | 29.89 | <0.0005 | <0.0005 |
| NSE: ≤12.5, >12.5 | 78 38 | 20 66 | 10.93 | 30.80 | <0.001 | <0.0005 |
| AGP: ≤1.40, >1.40 | 66 38 | 35 51 | 8.54 | 11.69 | <0.005 | <0.001 |
| Na: ≤135, >135 | 50 38 | 18 61 | 3.99 | 1.30 | <0.05 | NS |
| AP: ≤275, >275 | 50 38 | 52 30 | 1.57 | 5.65 | NS | <0.025 |
| CEA: ≤5.0, >5.0 | 48 40 | 48 38 | 2.23 | 4.46 | NS | <0.05 |

[a]: Median duration; N: Number examined; LRT: Log rank test; TT: Test for trend; NS: Not significant.

**Table III** Results of Cox's proportional hazards regression analysis in 86 patients with SCLC

| *Variable* | *Regression coefficient* | *SE* | *P* |
|---|---|---|---|
| NSE | 0.517 | 0.159 | <0.001 |
| PS | 0.408 | 0.130 | <0.001 |

The regression model is stratified into limited and extensive stage disease because of insufficient proportionality of death hazards between the two stages.

and their influence on survival (−0.38) indicates that the two variables may contain similar information and supports the role of NSE as an apparently important prognostic factor. Once NSE was included in he model, LDH did not provide additional significant information.

Significant prognostic influence of LDH has been confirmed by two recent series. Thus Østerlind & Andersen (1986) found major influence of LDH among 18 variables in 778 cases, and LDH was also among the six most important variables among 60 investigated features in a study on 407 patients (Cerny *et al.*, 1987).

Our CEA measurements were not related to survival. The conflicting results of the prognostic influence of CEA (Sculier *et al.*, 1985, Buccheri *et al.*, 1987) may be derived from differences in methods and variables included in the investigation, and we suspect that CEA has only inferior influence on the prognosis in SCLC.

We could not confirm the importance of AP described by Souhami *et al.* (1985) and by Vincent *et al.* (1987). None of the two studies did, however, include LDH in the analysis. Na, AGP, age and sex did not add critical information in this series of only 86 patients.

Patients with LD and ED did not have proportional death hazards and the Cox model therefore had to be stratified according to disease extent. The number of cases analysed in this series was regarded insufficient to establish a new prognostic index.

In conclusion, high levels of NSE and poor performance are of great importance for predicting the prognosis in SCLC. Compared to LDH, NSE values were increased in a greater fraction of our patients and therefore seems to be a more informative prognostic factor than LDH. It may therefore be reasonable to suggest that NSE should be included in future studies of prognostic factors and in clinical trials on SCLC.

**Figure 2** Scatter diagram of NSE with LDH. Correlation coefficient $r = 0.72$.

### References

ANDERSEN, P.K. & WAETH, M. (1984). Statistisk analyse af overlevelsesdata. FADL, Copenhagen.

AKOUN, G.M., SCARNA, H.M., MILLERON, B.J., BÉNICHOU, M.P. & HERMAN, D.P. (1985). Serum neuron-specific enolase. A marker for disease extent and response to therapy for small-cell lung cancer. *Chest*, **87**, 39.

BERKELEY, C.A. (1981). BMDP Statistical Software. University of California Press.

BUCCHERI, G.F., FERRIGNO, D., SARTORIS, A.M., VIOLANTE, B., VOLA, F. & CURCIO, A. (1987). Tumor markers in bronchogenic carcinoma. *Cancer*, **60**, 42.

CERNY, T., BLAIR, V., ANDERSON, H., BRAMWELL, V. & THATCHER, N. (1987). Pretreatment prognostic factors and scoring system in 407 small-cell lung cancer patients. *Int. J. Cancer*, **39**, 146.

COX, D.R. (1972). Regression models and life-tables. *J. Roy. Stat. Soc.* **34**, 187.

GANZ, P.A., BARAS, M., MA, P.Y. & ELASHOFF, R.M. (1984). Monitoring the therapy of lung cancer with alpha-l-acid glycoprotein. *Cancer Res.*, **44**, 5415.

LANZOTTI, V.J., THOMAS, D.R., BOYLE, L.E., SMITH, T.L., GEHAN, E.A. & SAMUELS, M.L. (1977). Survival with inoperable lung cancer. An integration of prognostic variables based on simple clinical criteria. *Cancer*, **39**, 303.

ØSTERLIND, K. & ANDERSEN, P.K. (1986). Prognostic factors in small cell lung cancer: Multivariate model based on 778 patients treated with chemotherapy with and without irradiation. *Cancer Res.*, **46**, 4189.

ØSTERLIND, K., PEDERSEN, A.G., VINDELØV, J. & 4 others (1986). Alternating or continuous chemotherapy of extensive stage small cell lung cancer (SCC). *Lung Cancer*, **2**, 127.

PEDERSEN, A.G., ØSTERLIND, K., VINDELØV, L., SØRENSEN, S., HANSEN, M. & DOMBERNOWSKY, P. (1987). Alternating or continuous chemotherapy of small cell lung cancer (SCC). A 3 armed randomized trial. *Proc. Am. Soc. Clin. Oncol.*, **6**, 185 (*abstract*).

PETO, R., PIKE, M.C., ARMITAGE, P. & 7 others (1977). Design and analysis of randomized clinical trials requiring prolonged observation of each patient, II. Analysis and examples. *Br. J. Cancer*, **35**, 1.

SCULIER, J.P., FELD, R., EVANS, W.K. & 4 others (1985). Carcinoembryonic antigen: A useful prognostic marker in small-cell lung cancer. *J. Clin. Oncol.*, **3**, 1349.

SOUHAMI, R.L., BRADBURY, I., GEDDES, D.M., SPIRO, S.G., HARPER, P.G. & TOBIAS, J.S. (1985). Prognostic significance of laboratory parameters measured at diagnosis in small cell carcinoma of the lung. *Cancer Res.*, **45**, 2878.

STANLEY, K.E. (1980). Prognostic factors for survival in patients with inoperable lung cancer. *J. Natl Cancer Inst.*, **65**, 25.

TARONE, R.E. (1975). Test for trend in life table analysis. *Biometrika*, **62**, 679.

VINCENT, M.D., ASHLEY, S.E. & SMITH, I.E. (1987). Prognostic factors in small cell lung cancer: A single prognostic index is better than conventional staging. *Eur. J. Cancer Clin. Oncol.*, **23**, 1589.

WHO HANDBOOK FOR REPORTING RESULTS OF CANCER TREATMENT (1979). World Health Organization. Publication No. 48, Geneva.

HISTOLOGICAL TYPING OF LUNG TUMOURS (1981). World Health Organization, Geneva.

ZELEN, M. (1973). Keynote address on biostatistics and data retrieval. *Cancer Chemother. Rep.* (part 3), **4**, 31.